# Long-term adherence to antiretroviral therapy in a South African adult patient cohort: a retrospective study

**DOI:** 10.1186/s12879-019-4410-8

**Published:** 2019-09-05

**Authors:** Atika Moosa, Tanuja N. Gengiah, Lara Lewis, Kogieleum Naidoo

**Affiliations:** 1 CAPRISA-Centre for the AIDS Programme of Research in South Africa, University of KwaZulu-Natal, Private Bag X7, Congella, Durban, 4013 South Africa; 2 MRC-CAPRISA HIV-TB Pathogenesis and Treatment, Research Unit, Nelson R Mandela School of Medicine, Doris Duke Medical Research Institute (2nd floor), University of KwaZulu-Natal, 719 Umbilo Road, Private Bag X7, Congella, Durban, 4013 South Africa

**Keywords:** Adherence, HIV, Antiretroviral therapy, Pill count, South Africa

## Abstract

**Background:**

South Africa has the highest HIV prevalence and supports the largest antiretroviral therapy (ART) programme globally. With the introduction of a test and treat policy, ensuring long term optimal adherence to ART (≥95%) is essential for successful patient and public health outcomes. The aim of this study was to assess long-term ART adherence to inform best practices for chronic HIV care.

**Method:**

Long-term ART adherence was retrospectively analysed over a median duration of 5 years (interquartile range [IQR]: 5.3–6.5) in patients initially enrolled in a randomised controlled trial assessing tuberculosis and HIV treatment integration and subsequently followed post-trial in an observational cohort study in Durban, South Africa. The association between baseline patient characteristics and adherence over time was estimated using generalized estimating equations (GEE). Adherence was assessed using pharmacy pill counts conducted at each study visit and compared to 6 monthly viral load measurements. A Kaplan Meier survival analysis was used to estimate time to treatment failure. The McNemar test (with exact *p*-values) was used to determine the effect of pill burden and concurrent ART and tuberculosis treatment on adherence.

**Results:**

Of the 270 patients included in the analysis; 54.8% were female, median age was 34 years (IQR:29–40) and median time on ART was 70 months (IQR = 64–78). Mean adherence was ≥95% for each year on ART. Stable patients provided with an extended 3-month ART supply maintained adherence > 99%. At study end, 96 and 94% of patients were optimally adherent and virologically suppressed, respectively. Time since ART initiation, female gender and primary breadwinner status were significantly associated with ≥95% adherence to ART. The cumulative probability of treatment failure was 10.7% at 5 years after ART initiation. Concurrent ART and tuberculosis treatment, or switching to a second line ART regimen with higher pill burden, did not impair ART adherence.

**Conclusion:**

Optimal long-term adherence with successful treatment outcomes are possible within a structured ART programme with close adherence monitoring. This adherence support approach is relevant to a resource limited setting adopting a test and treat strategy.

## Background

Currently, an estimated 36.7 million people are living with HIV (PLWH) worldwide [1]. South Africa has the highest adult HIV prevalence (18%) in sub-Saharan Africa and the largest adult population of PLWH in the world, estimated at approximately 7.5 million people [2, 3]. In September 2016, universal test and treat policy was incorporated into the South African national antiretroviral therapy (ART) guidelines and the country’s government funded national antiretroviral (ARV) programme is now the largest globally, providing HIV care and ART to over 4 million people [4, 5].

Access to ART is only one aspect of an effective HIV management programme. Optimal adherence to ART is essential and early studies reported that ≥95% adherence to ART was required to achieve and maintain viral suppression [6, 7]. Although recent studies have shown that virologic suppression may still be achieved with < 95% adherence levels, this is dependent on the ART regimen, duration of treatment and previous ART exposure [8, 9]. Furthermore, repeated adherence levels of < 100% and treatment interruptions are associated with an increased risk of nucleoside reverse transcriptase (NRTI) and non-nucleoside reverse transcriptase (NNRTI) resistance which form the backbone of current first line ART regimens in South Africa [10–12]. Poor adherence may lead to a number of adverse consequences on both individual and public HIV healthcare. Firstly, poorly adherent patients who acquire resistance to first line NNRTI-based regimens are switched to second and third line ARV regimens which are costlier, have a higher pill burden, increased dosing frequency and often have less tolerable side effects [13, 14]. Access to second line, third line and salvage treatment regimens are also limited or may be unavailable in developing countries [15]. Secondly, resistant HIV strains can be transmitted to others resulting in primary resistance to first line treatment regimens in newly infected ART-naïve patients or acquired resistance in infected patients already on ART [16, 17]. Thirdly, treatment failure due to non-adherence is associated with a greater risk of progression to Acquired Immune Deficiency Syndrome (AIDS) and mortality [18, 19]. In addition, hospital admissions due to HIV disease progression and opportunistic infections further add to the burden of public health costs in resource limited countries [20, 21]. Thus, it is still recommended that ≥95% adherence be maintained to achieve optimal viral suppression and prevent HIV resistance [22, 23].

Short term ART adherence rates (less than 2 years from treatment initiation) in South African patients from multiple urban HIV clinics is reported to range from 63 to 88% [24–27], however, there is a paucity of information on long-term adherence in this population. As ART access continues to expand with the implementation of test and treat, our public health facilities face the challenge of retaining an increasing number of PLWH in life time care with limited healthcare budgets and insufficient human resources [28]. Initial studies in African test and treat patient cohorts have reported > 80% adherence levels in the first year post-ART initiation [29, 30]. Monitoring and determining long term adherence patterns can provide insight into observed treatment outcomes and assist in developing adherence support and intervention strategies that would be relevant to resource-constrained countries implementing a test and treat policy.

## Methods

### Study aim, setting and design

The aim of this cohort study was to assess long-term adherence in a cohort of patients on ART for at least 5 years at the Centre for the AIDS Programme of Research in South Africa’s (CAPRISA) eThekwini Clinical Research site in Durban, South Africa.

Initially, HIV and tuberculosis (TB) co-infected patients were enrolled in the Starting Antiretroviral Therapy at Three Points in Tuberculosis (SAPiT) open label randomised controlled trial from June 2005 to July 2008 (*n* = 642). Details of the study outcomes have been published elsewhere [31, 32]. Baseline demographic data, including age, gender, education and socio-economic variables were obtained at enrolment. Study patients were initiated on a once daily, weight-based ART regimen containing efavirenz (EFV) or nevirapine (NVP) plus lamivudine (3TC) and enteric coated didanosine (ddI) either during or after completion of tuberculosis treatment. Viral load (Cobas® Amplicor HIV-1 Monitor, version 1.5, Roche) and CD4+ cell count (Becton Dickinson FACSCalibur™) measurements were conducted at screening, on randomization into the study and 6 monthly thereafter.

After completion of follow up in the SAPiT trial, patients were offered enrolment into a prospective observational study, TB Recurrence upon Treatment with HAART (TRuTH), investigating the rate of TB recurrence in HIV infected adults on ART who had completed pulmonary TB treatment (*n* = 402) [33, 34]. Viral load (Cobas® Ampliprep-Roche TaqMan®) and CD4+ cell count (Becton Dickinson FACSCalibur™) measurements were performed at least 6 monthly in the study. In 2011, ddI was replaced by tenofovir disoproxil fumarate (TDF) in viral suppressed patients in line with World Health Organisation (WHO) recommendations at the time [35]. Patients were enrolled into the TRuTH observational study from November 2009 to July 2011 and follow up was completed in 2014.

All patients received pre-ART education and ongoing adherence support counselling by trained counsellors and site pharmacists post-ART initiation. Pillboxes were provided to all patients as an adherence aid. Patients who did not come in on their scheduled clinic appointment date were telephonically or physically contacted. Close family members or friends were enlisted as treatment supporters, where possible, to provide additional social support for patients identified with adherence challenges.

The SAPiT and TRuTH studies were conducted under the oversight of the Biomedical Research Ethics Committee (BREC) at the University of KwaZulu-Natal (BREC ref.: E107/05 [SAPiT]; BF051/09 [TRuTH]). Participants in the SAPiT and TRuTH studies provided written informed consent. The current study is a retrospective secondary analysis of previously collected anonymized data from the parent trials with no direct participant contact (BREC ref.: BE046/16).

To assess long-term adherence, we conducted a retrospective analysis in a sub-cohort of patients on ART for at least 5 years from the time of ART initiation in the SAPiT trial until completion of follow up in the TRuTH study. To maintain continuity of care, patients accessed ART via the CAPRISA AIDS Treatment (CAT) programme at the same clinic after completion of follow up in SAPiT and before enrolment in TRuTH. Patients who never initiated ART, were lost to follow up in the SAPiT trial, did not receive ART from the site’s research pharmacy and for whom pill count data was missing for more than 6 consecutive months in either study were excluded.

### Long-term adherence assessment

Adherence to ART in both studies were determined from pharmacy pill count data and viral load measurements. Pill counts were conducted by the study pharmacist at monthly study visits in the SAPiT trial and at monthly or 3-monthly study visits in the TRuTH study. Adherence percentage was calculated using the following formula [36]:

$$ \frac{No. of\ pill s\ dispensed\  at\  previous\ visit- Number\ of\ pill s\ returned/ reported\ remaining/ lost\  at\  current\ visit}{Number\ of\ pill s\ that\ should\ have\ been\ ingested\ between\ visits\ \left( daily\ pill\ dose\ x\  no. of\ days\ between\ visits\right)}\times 100 $$

Optimal adherence was defined as ≥95% of doses taken in the time between the study visits [7]. Pill count data was not available for ART that was dispensed in the CAT programme (time period between exit form SAPiT study and enrolment into TRuTH study). Pill count adherence was not assessed for visits where there was a clinician-initiated treatment interruption or where pill count data was missing.

### Statistical analysis

Baseline demographic data were analyzed by descriptive statistics and reported as median with inter-quartile range (IQR) or proportions. Undetectable viral load was defined as < 400 copies/mL as per the South African national treatment guidelines at the time. In the SAPiT trial, treatment failure was defined as two HIV-1 RNA measurements of > 1000 copies/mL taken at least 4 weeks apart followed by ART discontinuation or change of all drugs in the ARV regimen. In the TRuTH study treatment failure was defined as two consecutive HIV-1 RNA measurement of > 1000 copies/mL at least 6 months apart followed by a change to a second line boosted lopinavir/ritonavir (LPV/r) based regimen.

Mean pill count adherence was calculated as the weighted mean of the monthly and/or 3-monthly adherence values over that time period, where the weights were calculated as the length of time between each study visit. The association between baseline patient characteristics and adherence over time was estimated using generalized estimating equations (GEE) with a logit-link function and exchangeable working correlation structure. A univariable analysis was first performed, and thereafter a multivariable model was estimated with gender, age and factors with a *p*-value < 0.2 in the unadjusted analyses. Interaction terms were incorporated into the multivariable model, one at a time, and were retained if found to be significant. Sensitivity, specificity, positive predictive value (PPV) and negative predictive value (NPV) (and associated 95% confidence intervals [95% CI]) were calculated to assess the extent to which mean pill count adherence (< 95% vs ≥95%) for each year on ART could be used to predict viral load outcomes (< 400 copies/mL vs ≥400 copies/mL) at the end of the same 12 month period. The McNemar test (with exact *p*-values) was used to determine the impact of concurrent ART and TB treatment on adherence to ART by analyzing changes in ART adherence and viral load suppression rates before and during TB treatment. The McNemar test was also applied to the adherence proportions and viral load suppression rates before and after switching to a second line regimen to assess the impact of the pill burden on adherence. A Kaplan Meier survival analysis was used to estimate time to treatment failure. All statistical tests were conducted at 5% level of significance. Statistical Analysis System (SAS) version 9.4 software was used to conduct statistical analysis.

## Results

**Fig. 1:**
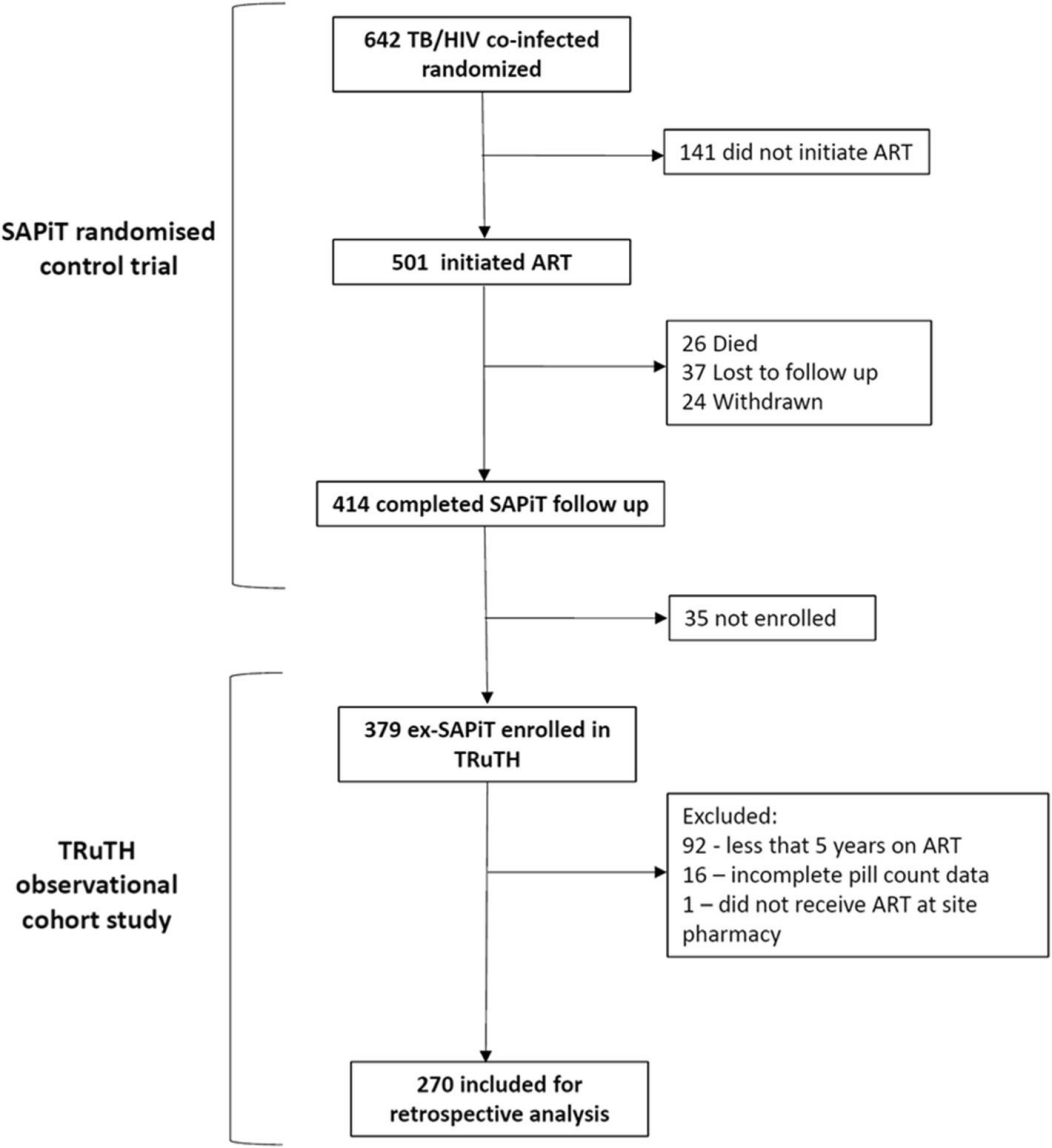
Selection of patient cohort on ART for at least 5 years

A total of 270 patients met the inclusion criteria for this analysis (Fig. 1). The median age was 34 years (IQR:29–40) and 54.8% were female (Table 1). At ART initiation the median CD4+ cell count was 145 cells/mm^3^ (IQR:76–249) and median viral load was 141,000 copies/mL (IQR:37757–386,000). Most patients (254/270) were classified as WHO stage 3 at baseline. A third of the cohort had completed secondary school, 60% were in full or part-time employment and more than half of all patients had disclosed their HIV status. The median duration on ART from initiation to completion of follow up was 70 months (IQR: 64–78).

**Table 1:** Baseline demographics and clinical characteristics

Variable	Total (*n* = 270)
Demographics	
Age (years), median (IQR)	34 (29–40)
Female (%)	148 (54.8)
Education^a^	
Primary school or less (%)	58 (21.5)
Secondary school not complete (%)	123 (45.6)
Secondary school complete (%)	88 (32.6)
Head of household (%)	179 (66.3)
Primary breadwinner (%)^a^	190 (70.4)
Stable partner (%)	46 (17.0)
Employed, full or part time (%)	164 (60.7)
Access to tap water and electricity (%)^a^	236 (87.4)
Tested for HIV prior to enrolment (%)^b^	134 (49.6)
Disclosed HIV status (%)^b^	154 (57.0)
Clinical Characteristics	
CD4+ count (cells/mm^3^)^c^, median (IQR)	145 (75–249)
Viral Load (copies/mL)^cd^, median (IQR)	141,000 (37757–386,000)
WHO Stage	
Stage 3 (%)	254 (94.1)
Stage 4 (%)	16 (5.9)

^a^ 1 missing data

^b^ 9 missing data

^c^ 4 missing data

^d^ measured at visit prior to or at initiation of ART

The time period between patient exit from SAPiT and enrolment in TRuTH (where no pill count data available) was a median of 13 months (IQR: 5–21). The number of patients for whom pill count data was available in each 6 month period is shown in Table 2. Mean adherence was ≥95% for each year on ART. The majority of the cohort (94.8%) had an overall mean ART adherence of ≥95%. Of the 14 patients with sub-optimal adherence, nine had adherence estimates in the 90–95% range, two in the 80–90% range, two in the 70–80% range, and only one had less than 70% adherence. Mean adherence peaked (99.3%) at 18–24 months after ART initiation and remained above 98% from 32 months onward.

**Table 2:** Mean adherence to ART by pill count

Time on ART, *months*	No. of patients	Mean adherence, *% (SE)*
≤6	268	97.7 ((0.32)
> 6–12	270	98.3 (0.38)
> 12–18	258	99.1 (0.14)
> 18–24	205	99.3 (0.13)
> 24–30	115	99.0 (0.33)
> 30–36	161	98.8 (0.41)
> 36–42	201	99.2 (0.24)
> 42–48	243	99.0 (0.31)
> 48–54	260	98.7 (0.36)
> 54–60	268	98.7 (0.26)
> 60–66	270	98.8 (0.27)
> 66–72	172	98.6 (0.49)
> 72	116	98.3 (0.69)

*SE* Standard error

**Fig. 2:**
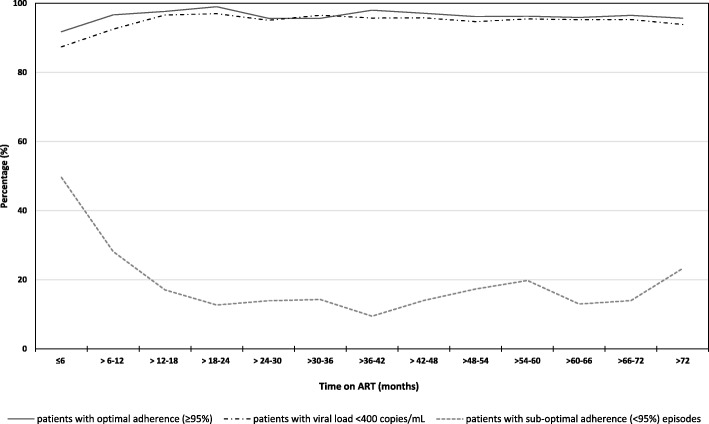
Patient adherence and viral load suppression over duration of follow up period

In the 6 months post-ART initiation, 91.8% of patients had optimal adherence. Half of all patients had at least one sub-optimal adherence measurement (< 95%) in the first 6 months after starting ART, less than 20% between the first and sixth year and 23% after 6 years on ART (Fig. 2). In the TRuTH study, patients with optimal adherence by pill count and an undetectable viral load (*n* = 267) were provided with an extended 3-month ART supply at a total of 1061 study visits. Overall mean adherence for patients who received a 3-month ART package was 99.5%.

Patient variables included in the univariable and multivariable GEE analyses are shown in Table 3. In the multivariable model, time since ART initiation (adjusted odds ratio [aOR] =1.10; 95%CI: 1.05–1.16), female gender (aOR = 1.33, 95%CI:1.05–1.67) and being a primary breadwinner (aOR = 1.32, 95%CI 1.02–1.72) were significantly associated with achieving optimal adherence.

**Table 3:** Factors associated with optimal adherence (≥95%) to ART

Variable	OR	95%CI	*p*-value	aOR	95%CI	*p*-value
Time since ART initiation (years)	1.10	1.05–1.16	0.0002	1.10	1.05–1.16	0.0001
Gender (ref: male)						
Female	1.25	0.99–1.58	0.055	1.33	1.05–1.67	0.017
Age (ref: > 40 years)						
≤25 years	0.78	0.51–1.19	0.249	0.77	0.46–1.27	0.302
26–40 years	1.06	0.83–1.36	0.631	1.11	0.85–1.43	0.450
Marital status (ref: not married)						
Married	0.91	0.69–1.21	0.537			
Education^a^ (ref: Secondary school completed)						
No schooling	2.39	0.91–6.29	0.078	2.42	0.95–6.16	0.063
Secondary school not completed	0.91	0.71–1.15	0.422	0.92	0.72–1.19	0.535
Employment status (ref: student)						
Employed	0.72	0.42–1.25	0.242	0.70	0.42–1.17	0.170
Unemployed	0.63	0.35–1.11	0.110	0.66	0.39–1.12	0.124
Primary breadwinner^a^ (ref: no)						
Yes	1.20	0.92–1.56	0.177	1.32	1.02–1.72	0.034
Household tap water^a^ (ref: no)						
Yes	0.85	0.52–1.38	0.515			
Household electricity^a^ (ref: no)						
Yes	0.83	0.55–1.25	0.370			
Telephone^a^ (ref: no)						
Yes	1.19	0.92–1.53	0.189	1.20	0.93–1.54	0.162
Tested for HIV prior to study^b^ (ref: no)						
Yes	0.98	0.77–1.24	0.869			
Disclosed HIV status^b^ (ref: no)						
Yes	1.08	0.85–1.37	0.521			
Baseline CD4 count (cells/mm^3^)^c^	1.00	0.9986–1.0004	0.284			
Baseline WHO stage (ref: Stage 3)						
Stage 4	0.85	0.49–1.47	0.553			

*ART* Antiretroviral therapy

*OR* Odds ratio

*aOR* Adjusted odds ratio

*CI* Confidence interval

^a^ 1 missing data

^b^ 9 missing data

^c^ 4 missing data

Viral suppression rates were 87.4% 6 months after ART initiation and thereafter increased and remained above 92% throughout follow up (Fig. 2). Eleven patients had a viral load > 400 copies/mL after 5 years and 94% of patients were virologically suppressed at the end of the follow up period. Pill count showed high sensitivity in predicting viral suppression but had poor specificity when the viral load was detectable (Table 4).

**Table 4:** Performance of pill count adherence in determining viral load suppression

Pill count adherence	Viral load unsuppressed (≥400 copies/ml)	Viral load suppressed (< 400 copies/ml)	Sensitivity (95% CI)	Specificity (95% CI)	PPV (95% CI)	NPV (95% CI)
Year 1 on ART (*n* = 268)						
< 95%	2 (1%)	9 (3%)	97% (94–98)	22% (3–60)	97% (94–99)	18% (2–52)
≥ 95%	7 (3%)	250 (93%)				
Year 2 on ART (*n* = 201)						
< 95%	1 (1%)	4 (2%)	98% (95–99)	10% (0–45)	95% (91–98)	20% (1–72)
≥ 95%	9 (4%)	187 (93%)				
Year 3 on ART (*n* = 166)						
< 95%	3 (2%)	4 (2%)	97% (94–99)	33% (7–70)	96% (92–99)	43% (10–82)
≥ 95%	6 (4%)	153 (92%)				
Year 4 on ART (*n* = 243)						
< 95%	0	4 (2%)	98% (96–100)	0	95% (91–97)	0
≥ 95%	13 (5%)	226 (93%)				
Year 5 on ART (*n* = 233)						
< 95%	1 (1%)	11 (5%)	95% (91–98)	9% (0–41)	95% (92–98)	8% (0–38)
≥ 95%	10 (4%)	211 (90%)				

*CI* Confidence interval

*ART* Antiretroviral therapy

*PPV* Positive predictive value

*NPV* Negative predictive value

*n* Number of patients

**Fig. 3:**
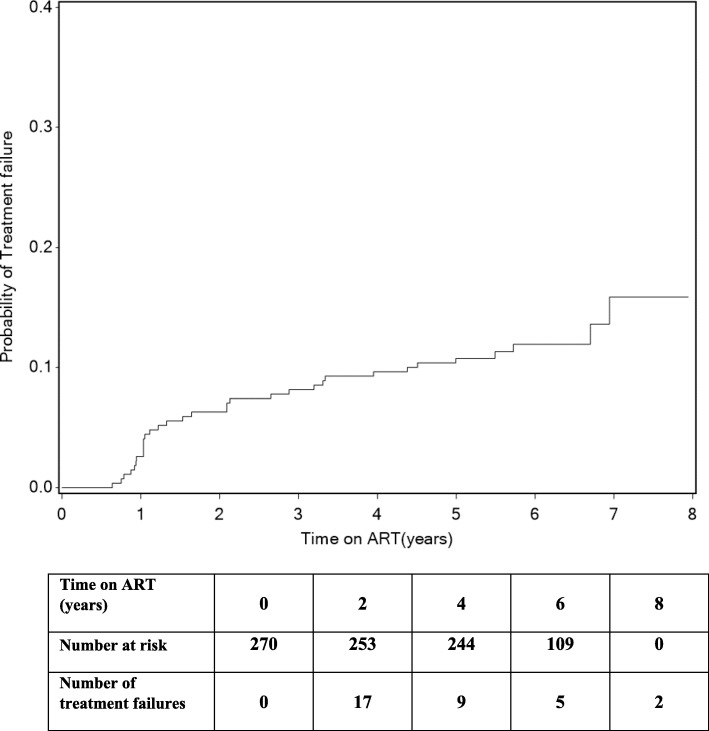
Probability of treatment failure over time

Thirty-three patients experienced treatment failure with approximately half of all treatment failures (*n* = 17) occurred within 2 years after ART initiation. The cumulative probability of treatment failure was 10.7% at 5 years after ART initiation (Fig. 3). Adherence and viral load measurements prior to and after switching to a second line boosted LPV/r regimen was available for 21 of the treatment failure patients. The number of patients with optimal adherence improved and there was a significant increase in viral load suppression at 6 and 12 months after switching to the second line regimen (Table 5).

**Table 5:** Adherence and viral load suppression in HIV-TB co-infected patients and patients on second-line ART

	HIV-TB co-infected patients		Patients switched to second-line ART			
	6 month period before TB treatment, *n (%)*	During TB treatment, *n(%)*	6 month period before regimen change, *n(%)*	6 months after regimen change, *n(%)*	6 month period before regimen change, *n(%)*	12 months after regimen change^a^, *n(%)*
Mean ART adherence						
< 95%	0 (0)	3 (18)	7 (33)	2 (10)	7 (33)	0
≥ 95%	17 (100)	14 (82)	14 (67)	19 (90)	14 (67)	19 (90)
*p*-value	0.250		0.180		0.016	
Viral load						
≥ 400 copies/mL	2 (12)	3 (18)	21 (100)	5 (24)	21 (100)	1 (5)
< 400 copies/mL	15 (88)	14 (82)	0 (0)	16 (76)	0 (0)	19 (90)
*p*-value	0.563		< 0.0001		< 0.0001	

*n* Number of patients

*ART* Antiretroviral therapy

*TB* Tuberculosis

^a^ 3 missing data

Of the 21 patients diagnosed and initiated on treatment for recurrent TB in the TRuTH study, three exited the study whilst on TB treatment and one did not complete their course of TB treatment. In the remaining 17 patients, there was a decline in optimal adherence and viral load suppression when patients were on both ART and TB treatment, but this was not statistically significant (Table 5).

## Discussion

Our study found overall high adherence to ART, determined by pill count, in this South African cohort followed up over a period of more than 5 years. Treatment outcomes were successful on first and second line treatment regimens, with 93.9% of patients virologically suppressed at study completion and only 12% experiencing treatment failure over the entire study period. The likelihood of achieving optimal adherence also improved with time on ART. Adherence during the first year of treatment was higher (98%) than those reported in several African studies, where adherence estimates ranged from 72 to 94% [25, 37–41]. Over the long term, mean adherence was maintained ≥95% for each year of follow up. Long-term adherence studies in Botswana, Senegal and Nigeria also found comparably high adherence levels [42–45]. Intensive pre-ART education and counselling sessions, an ongoing adherence support programme post-ART initiation, provision of pillboxes and use of a once-daily ART regimen, support measures that have been shown to assist adherence, may have played a role in helping to attain high adherence [46–49]. Additionally, enteric coated ddI was used instead of stavudine (D4T) in our first line ART regimen due its better side-effect profile, thereby avoiding non-adherence and discontinuation associated with stavudine toxicity observed elsewhere [50, 51].

Female patients were 33% more likely to have optimal adherence compared to males in our cohort. Although gender has not been consistently found to influence ART adherence, HIV infected women in developing countries tend to demonstrate better health seeking behaviours than men which may be motivated by their role as the primary care giver in their families [52, 53]. Being a primary breadwinner was also positively associated with optimal adherence which could indicate that the responsibility of being the main income earner may have been an adherence facilitator in this group of patients.

Viral suppression rates exceeded 90% for each year throughout the observation period. Similar long-term virologic outcomes have been reported in other low- and middle-income regions [51, 54–58]. The proportion of patients failing treatment was highest in the 2 years after ART initiation, thereafter, the number of treatment failures declined with each subsequent year on ART. Interestingly, non-adherence by pill count was most frequent in the first 6 months of ART in our cohort. Poor adherence in the initial months after ART initiation has been shown to increase the risk of virologic failure in later years and these early adherence lapses may be the reason for the higher number of treatment failures initially observed thus highlighting the importance of prioritizing adherence monitoring and support interventions to ensure optimal adherence from ART initiation [25, 44, 59–61].

Sensitivity was high, but specificity was low for the use of pill count data as a proxy for viral load outcomes. Whilst some studies have demonstrated an association between pharmacy adherence measures and virologic outcomes others have found poor agreement between the two measures [39, 40, 62–65]. We used an adherence threshold of ≥95%, however, viral suppression has been reported in patients with 85–94% adherence on long-term ART [66]. Furthermore, while pill counts provide a more objective method of adherence assessment, patient manipulation is possible as non-adherent patients may discard or leave behind medication not taken and return the desired number of pills to demonstrate good adherence [67, 68]. Although plasma viral suppression is possible with sub-optimal adherence, low-level viral replication continues in the plasma and reservoirs such as the central nervous system and genital tract, the long-term effects of which have not been fully elucidated [8, 69]. Therefore, high adherence to ART remains essential to ensuring the best clinical outcomes.

Patients who were provided with an extended 3-month ART supply in the TRuTH study were able to maintain high adherence levels over the extended time between clinic visits. This finding provides reassurance that stable patients can maintain optimal adherence when provided with an extended ART supply and patients who successfully adhere to treatment in the first 2 years are ideal candidates for more than 1 month’s supply of treatment. In an overburdened public health care setting, such as South Africa, this practice can ease clinic patient load and reduce the need for monthly clinic visits which impact negatively on patient finances and employment prospects.

High pill burden, regimen complexity and dosing more than once a day are known to hinder adherence [70]. Despite the additional pill burden and dosing frequency with switching from a once daily NNRTI-based regimen to a twice daily LPV/r regimen, with almost double the number of pills to be taken, adherence was not impaired and viral load suppression improved significantly in our patients who failed first-line treatment. The use of a boosted LPV/r regimen, provision of enhanced adherence counselling to patients on the implications of treatment failure with reinforcement of the importance of good adherence and enlisting a treatment supporter, where possible, may account for the improved outcomes [71–73].

HIV and TB co-infection rates are highest in South Africa [74]. Concurrent treatment of both diseases reduces morbidity and mortality; however, patients must cope with the additional pill burden and overlapping side effects when taking ART and TB treatment which can lead to impaired or selective adherence to either ART or TB medication [32, 75, 76]. We found no significant changes in ART adherence or viral suppression in our patients on concurrent ART and TB treatment. This is commensurate with findings from other South African studies [77].

There are several limitations to our study. This was a retrospective study of ART adherence conducted at a single research site and adherence behaviour may have been influenced by participation in a clinical trial, where patients were routinely monitored and received extensive adherence support and counselling. Factors associated with adherence behaviour were limited to available baseline patient and clinical data. In addition, our patients were initiated on ART at lower CD4 counts with advanced HIV disease and sicker patients may be more motivated to adhere to treatment [78]. Hence, adherence in this cohort may not be generalizable to a ‘test and treat’ or public health patient setting. Assessment of adherence by pill count may be prone to bias as non-adherent patients familiar with the pill count assessment may ‘count pills’ and return the required number of pills to show good adherence. An accurate adherence calculation is not possible for patients who did not bring any remaining pills back, reported lost pills and/or pills left at home. The exclusion of patients that were lost to follow up, patients with missing pill count data for more 6 consecutive months, and the lack of pill count data in the time period between the SAPiT and TRuTH studies, could have resulted in an overestimation of adherence in our cohort.

Optimal long-term adherence rates observed in this cohort support the evidence of high adherence reported in other long-term African patient cohorts. Pill count should not be used as a standalone measure of adherence as it was not a good predictor of viral failure and viral load measurement remains the benchmark for monitoring treatment response. However, adherence during the 6 months post-ART initiation period has been shown to impact long-term treatment outcomes and, per in-country treatment guidelines, viral load is only measured 6 months after ART initiation and thereafter annually. Therefore, we recommend adherence monitoring using pill count as a quick, simple adherence measure at each visit in this critical 6 month period to identify patients with early adherence challenges and allow for timeous intervention.

Provision of adherence support measures which may have assisted patient adherence included individual and group counselling, provision of pill boxes, treatment supporters and tracking of patients who missed their clinic appointment date. Pharmacists were also able to identify patients experiencing adherence challenges when conducting pill counts and could either provide or refer patients for appropriate adherence counselling. We also demonstrated that providing stable patients with a 3-month ART supply was feasible as optimal adherence was still maintained over this extended period. This has the potential to reduce clinic workloads and patient time and costs associated with monthly clinic visits and is currently being investigated further in a randomized control trial [79]. Several studies are currently being undertaken in sub-Saharan Africa to monitor long term treatment outcomes in tests and treat populations [80]. Further research on long term adherence and adherence support strategies in these test and treat cohorts would help to determine if these factors could support optimal long term adherence to ART.

## Conclusion

In summary, our study found optimal long-term adherence with good treatment outcomes in a structured ART programme with close adherence monitoring and support. With the implementation of a test and treat policy and rapid scale up of ART provision, it is imperative that patient adherence is monitored early on from ART initiation and then continually assessed and supported, to ensure a successful ART programme in South Africa.

## Data Availability

The data for this study can be made available by the corresponding author upon reasonable request.

## Abbreviations

3TC: Lamivudine
AIDS: Acquired Immune Deficiency Syndrome
ART: Antiretroviral therapy
ARV: Antiretroviral
BREC: Biomedical Research Ethics Committee
CAPRISA: Centre for the AIDS Programme of Research in South Africa’s
CAT: CAPRISA AIDS Treatment
D4T: Stavudine
ddI: Didanosine
EFV: Efavirenz
IQR: Inter-quartile range
LPV/r: Lopinavir/ritonavir
NNRTI: Non-nucleoside reverse transcriptase
NRTI: Nucleoside reverse transcriptase
NVP: Nevirapine
PLWH: People are living with HIV
SAPiT: Starting Antiretroviral Therapy at Three Points in Tuberculosis
SD: Standard deviation
TB: Tuberculosis
TDF: Tenofovir disoproxil fumarate
TRuTH: TB Recurrence upon Treatment with HAART
WHO: World Health Organisation
